# Effects of High-Fat Diet at Two Energetic Levels on Fecal Microbiota, Colonic Barrier, and Metabolic Parameters in Dogs

**DOI:** 10.3389/fvets.2020.566282

**Published:** 2020-09-25

**Authors:** Alex Moinard, Cyrielle Payen, Khadija Ouguerram, Agnès André, Juan Hernandez, Amandine Drut, Vincent C. Biourge, Jan S. Suchodolski, John Flanagan, Patrick Nguyen, Véronique Leray

**Affiliations:** ^1^Nutrition, PhysioPathology and Pharmacology Unit (NP3), Oniris, College of Veterinary Medicine, Food Sciences and Engineering, CRNH, Nantes, France; ^2^UMR 1280 Physiopathology of Nutritional Adaptations (PhAN), INRAE, CRNH, West Human Nutrition Research Center, CHU, Nantes, France; ^3^USC 1383 Cellular and Molecular Immunoendocrinology (IECM), INRAE, Oniris, College of Veterinary Medicine, Food Sciences and Engineering, Nantes, France; ^4^Research Center, Royal Canin SAS, Aimargues, France; ^5^Gastrointestinal Laboratory, Texas A&M University, College Station, TX, United States

**Keywords:** high-fat diet (HFD), microbiota (microorganism), colonic barrier, insulin sensitivity, dog

## Abstract

Increased consumption of energy-rich foods is a key factor in overweight, obesity, and associated metabolic disorders. This would be, at least in part, related to microbiota disturbance. In rodent models of obesity, microbiota disruption has been associated with alteration of the intestinal barrier, endotoxemia, inflammation grade, and insulin sensitivity. The aim of the present study was to assess the effects of a high-fat diet (HFD), fed at two energetic levels, on microbiota, intestinal barrier, and inflammatory and metabolic parameters in dogs. A HFD (33% fat as fed, 4,830 kcal/kg) was given to 24 healthy Beagle dogs at 100% (HF-100; *n* = 8) and at 150% (HF-150; *n* = 16) of their maintenance energy requirements for 8 weeks. Analysis of similarity revealed a significant difference in gut microbiota β-diversity following the diet compared to week 0 in both groups while α-diversity was lower only in the HF-150 group. Firmicutes/Bacteroidetes ratio was higher in the HF-150 group compared to the HF-100 group at weeks 2 and 8. A reduction in insulin sensitivity was observed over time in the HF150 group. Neither endotoxemia nor inflammation was observed in either group, did not find supporting data for the hypothesis that the microbiota is involved in the decline of insulin sensitivity through metabolic endotoxemia and low-grade inflammation. Colonic permeability was increased at week 4 in both groups and returned to initial levels at week 8, and was associated with modifications to the expression of genes involved in colonic barrier function. The increase in intestinal permeability may have been caused by the altered intestinal microbiota and increased expression of genes encoding tight junction proteins might indicate a compensatory mechanism to restore normal permeability. Although simultaneous changes to the microbiota, barrier permeability, inflammatory, and metabolic status have not been observed, such a causal link cannot be excluded in dogs overfed on a HFD. Further studies are necessary to better understand the link between HFD, intestinal microbiota and the host.

## Introduction

Despite normal temporal variations, it has been shown that, in a given environment, the fecal microbiota can be considered stable, in dogs (1) as in other species (2, 3). However, numerous environmental factors such as the diet can, transiently or persistently, change the microbiota. In dogs, the intestinal microbiota can be shifted by both high-protein diets (4–6) and the variation in the protein to carbohydrate ratio (7, 8). As in other species, fermentable fiber also plays a role in the intestinal microbiota (6, 9–11). Lastly, a HFD (12) as well as the variation in the fat-to-carbohydrate ratio change the intestinal microbiota (13).

Canine obesity is associated with many metabolic and hormonal disturbances, such as a lower insulin sensitivity. We have previously shown that weight gain in overfed dogs was associated with decreased insulin sensitivity, low-grade inflammation, and altered lipid status (14, 15). Conversely, weight loss in overweight dogs resulted in an improvement in insulin sensitivity, a decrease in pro-inflammatory cytokines (16) and an improvement in lipid profile (17).

Within this given environment, different physiological or pathological states determine differences in the intestinal microbiota. In particular, it has been shown that the microbiota of lean individuals or animals differs from that of obese ones. In humans and mice the main difference concerns an increase in the ratio of the two predominant phyla in these species, Firmicutes and Bacteroidetes (18, 19). Compared with lean mice and regardless of kinship, ob/ob animals have a 50% reduction in the abundance of Bacteroidetes and a proportional increase in Firmicutes (18). In humans, the relative proportion of Bacteroidetes is decreased in obese people by comparison with lean people, and that this proportion increases with weight loss on two types of low-calorie diet (19). Differences in the intestinal microbiota have also been described in obese dogs compared with lean dogs (12, 20, 21). However, the heterogeneity of the results did not allow to identify a bacterial signature of obesity in dogs as has been done in humans and mice (18, 19). Interestingly it has also been reported that the effect of the diet on the microbiota could be influenced by the dog's body condition score (BCS), used as an index of adiposity (5).

Many studies have suggested that the intestinal microbiota could participate in the development of obesity. Among the proposed mechanisms, the microbiota could enhance energy harvest from the diet by increasing the monosaccharide uptake in the small intestine (22) and the production of short-chain fatty acids (SCFA) through fermentation in the hindgut (23). Nevertheless, the role of SCFA toward obesity, as well as the reasons for enhanced fecal concentrations of SCFA are controversial (24).

The gut microbiota could also play a role in promoting obesity by eliciting an endotoxemia, due to systemic circulation of lipopolysaccharides (LPS), a constituent of intestinal gram-negative bacteria walls. This assertion is based on the observation that, in mice, a chronic infusion of LPS triggers similar increase of whole-body, hepatic, and adipose tissue mass, and similar metabolic effects, notably hyperglycemia and hyperinsulinemia, to that of a HFD (25). Also, it has been shown that a HFD may induce an increase in intestinal LPS-containing bacteria, and an alteration of the intestinal barrier by a mechanism associated with a reduced expression of epithelial tight junction proteins (26). Both the changes in the microbiota and in intestinal permeability could lead to endotoxemia, and contribute to the development of obesity.

In studies that have investigated the consequences of HFD on gut microbiota, it was difficult to distinguish the effects driven by increased calorie intake from those driven by increased body weight (BW) (and increased body fat) because the HFD was usually administered at a hyperenergetic level (or even *ad libitum*). We also wanted to put forward the hypothesis of Cani et al. (26), according to which the microbiota would be involved in the decrease of insulin sensitivity through metabolic endotoxemia and low-grade inflammation accompanied by an increase in intestinal permeability. The HFD would induce changes in the intestinal microbiota involved in the development of weight gain and alterations of the intestinal barrier and metabolic and inflammatory parameters. The current study was designed to compare the effects of a HFD fed at maintenance energy requirements to those of the same diet at 150% maintenance on the microbiota, the intestinal barrier and the host physiology (inflammatory and metabolic variables).

## Materials and Methods

### Animals and Housing

Twenty-four healthy female spayed Beagle dogs (age: 5.1 ± 0.4 years, mean ± SEM; BW: 13.2 ± 1.3 kg; BCS: 6/9 (range, 5/9–6/9) took part in this study. Sample size was not calculated formally. Instead, inclusion of 24 dogs was based on the capacity of Oniris' facilities to guarantee animal welfare and to ensure that the sampling workload could be conducted in reliable conditions by one person in order to avoid manipulation bias.

Dogs were housed in pairs based on their social compatibility, in an outdoor enclosure that included a sheltered place to sleep (2.0 x 5.0 m). They had daily interaction with their caregivers and daily access to outside playgrounds. Dogs were vaccinated yearly and dewormed bi-annually. They were declared healthy on the basis of a clinical examination, blood cell count and serum biochemistry. Dogs had received no medications expected to alter the gut microbiota (e.g., antibiotics) over the previous 2 months.

The dogs were housed at ONIRIS, the National Veterinary School of Nantes, France, according to animal welfare regulations of the French Ministry of Research. The protocol fulfilled European Union guidelines on animal experimentation (directive 2010/63 on the protection of animals used for scientific purpose) and was approved by both Royal Canin's Ethical Review Committee (090118-03) and the Ethics Committee of “Pays-de-la-Loire” (Apafis#13800).

### Diets and Study Design

Prior to starting the study, the dogs had been fed with a standard maintenance diet for 5 months (Medium Adult, Royal Canin, Aimargues, France; Table 1). During the study, all dogs received a hyperlipidic, normoproteic commercial dry diet for 8 weeks (33% fat, 29% protein, 4,830 kcalME (metabolizable energy)/kg (Marathon 5000® Royal Canin, Aimargues, France; Table 1). Dogs were randomly allotted to two groups: the HF-100 group which were given the diet at 100% of maintenance energy requirement (*n* = 8; 103 ± 11 kcalME/kgBW^0.75^/day) and the HF-150 group which ate the diet at 150% of maintenance energy requirement (*n* = 16; 168 ± 26 kcalME/kgBW^0.75^/day). The food was given once a day, and water was constantly available. The quantity of diet necessary to meet the maintenance requirement was calculated from the energy that had been necessary to maintain constant weight in the pre-study period.

**Table 1:** Composition of the pre-study diet and high-fat diet on an as-fed and per-energy basis.

	**Pre-study diet**	**High-fat diet**
	**%**	**%**
Water	9.5	6.1
Fat	16.0	33.1
Protein	23.0	29.3
Starch	38.4	13.4
Total fiber	6.7	10.1
Ash	6.4	8.0
ME (kcal/100g)	357.0	483.0
	**%Energy**	**%Energy**
Fat	34%	63%
Protein	30%	25%
Starch	36%	12%

**Figure 1:**
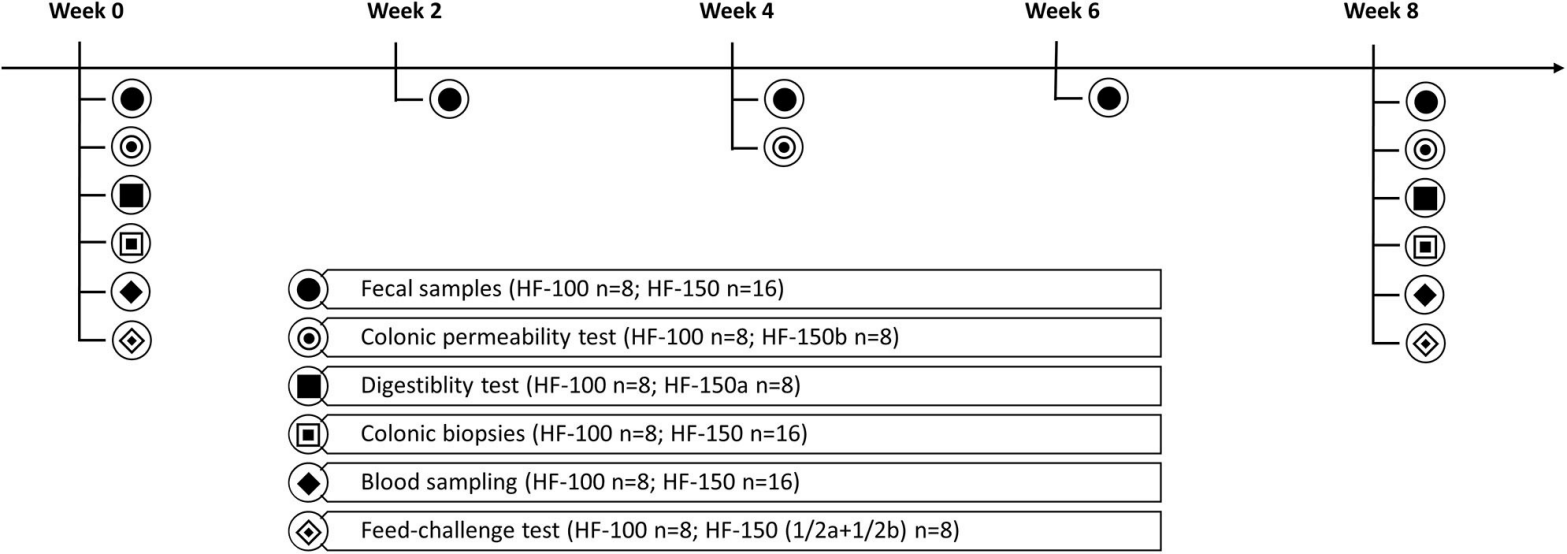
Experimental design of the study. HF-100 corresponds to the group of dogs fed the high-fat diet at 100% of maintenance energy requirement and HF-150 corresponds to the group of dogs fed the high-fat diet at 150% of maintenance energy requirement. HF-150a and HF-150b are two separate subgroups consisting of 8 dogs from the HF-150 group.

The design of the study is shown in Figure 1. BW and BCS were recorded each week. Blood samples were carried out after a 24-h unfed period, and postprandially following a feed challenge test, before and at the end of 8-week period. Colonic biopsies were performed, and diet digestibility was determined, according to the same time-schedule. Colonic permeability was determined at baseline, and after 4 and 8 weeks. Fresh fecal samples were collected at baseline then every two weeks.

### Digestibility

Organic matter, crude protein, and fat matter digestibility was determined according to FEDIAF Nutritional Guidelines (27).

### Feed-Challenge Test

After a 24-h unfed period, dogs were exposed to a feed challenge test. The oral challenge-feed was composed of 3 g/kgBW^0.75^ protein, 3 g/kgBW^0.75^ glucose, 2.5 g/kgBW^0.75^ lipids. Blood samples were taken from the jugular vein before and 30, 60, 90, 120, 150, 180, 240, and 360 min after the challenge-feed administration. AlphaTRAK, a validated portable canine blood glucose meter (Abbott Animal Health, Abbott Park, IL, USA), was used to assay blood glucose concentration immediately after collection. Plasma samples were stored at −80°C until assays were performed.

### Serum and Plasma Assays

Leptin (Millipore Corporation, Billerica, MA, USA), adiponectin (Wuhan Fine Biotech Co, Wuhan, China), ghrelin (BioVendor, Karasek, Czech Republic), gastric inhibitory polypeptide (GIP), and neuropeptide Y (NPY) (BlueGene Biotech, Shanghai, China), C-reactive protein (CRP) (Helica, Santa Ana, CA, USA), serum amyloid A (SAA) (Abcam, Cambridge, UK) and haptoglobin (Abcam, Cambridge, UK) concentrations were measured in plasma by specific enzyme linked immunosorbent assay (ELISA) kits according to manufacturers' instructions. Insulin concentration was determined by radioimmunoassay (Insulin IRMA KIT, Beckman Coulter, Nyon, Switzerland). Plasma LPS concentration was measured with a limulus amoebocyte lysate (LAL) assay using pyrochrome chromogenic reagent (Associates of Cape Cod, INC., East Falmouth, MA, USA). Plasma lipopolysaccharide binding protein (LBP) concentration was assayed using an ELISA kit (Novatein Biosciences, Woburn, MA, USA). Superoxyde dismutase (SOD), glutathione peroxidase (GPx), and total antioxidant status (TAS) were assayed by colorimetric method (Randox, UK). Triglycerides (TG), total cholesterol, HDL-cholesterol (HDL-C), and non-esterified fatty acids (NEFA) were determined by colorimetric assays (Sobioda, Montbonnot Saint Martin, France).

### Colonic Permeability

#### Sugar Ingestion and Urine Collection

After a 24-h unfed period, BW was recorded and dogs were placed in individual collection pens. Water was continuously available *ad libitum*. Two milliliters per kg BW of a sugar solution (150 mg/ml of lactulose (Mylan Pharma, Saint-Priest, France) and 100 mg/ml of sucralose (Sigma-Aldrich Co, St Louis, MO, USA)) was administered orally, as previously described in dogs (28). Four hours after the administration of sugars, dogs were fed their daily ration. Forty-eight-hour urine was collected and samples stored at −20°C for subsequent analysis.

#### Urine Sugar Content

The preparation of urine samples was made as previously described by Hernot et al. except for the internal standard, turanose (28). The concentration of each sugar was assessed by gas chromatography-mass spectrometry technique (Agilent technologies, Santa Clara, United States). The urine sugar concentration and the volume of 48-h collected urine were used to calculate the total amount of excreted sugars. The amount of each sugar in urine was expressed as a percentage of the ingested amount. Colonic permeability was estimated by the ratio of %lactulose to %sucralose (L:S).

### Biopsies

After a 24-h unfed period and two colonic preparations (the day before and immediately before colonoscopy) by warm enema using water, six biopsies of colon mucosa per animal at both sampling times (0 and 8 weeks) were performed using a fiberscope, between 15 and 30 cm from the anus, under anesthesia. Tissue biopsies were snap frozen immediately in liquid nitrogen and kept at −80°C, pending RNA tissue extraction.

Preanesthetic medication included medetomidine (Domitor®, Elanco, Neuilly sur Seine, France) at 15 μg/kgBW and butorphanol (Dolorex®, MSD Animal Health, Beaucouze, France) at 0.2 mg/ kgBW, and was administered as an IV bolus 10 min prior to anesthetic induction. General anesthesia was induced with propofol (Propovet®, Axience, Pantin, France) at 2 mg/kgBW intravenously. After orotracheal intubation, general anesthesia was maintained with isoflurane in oxygen. Lactated Ringer's solution (Virbac®, France) was administered intravenously at 5 ml/kgBW/h. After completion of the procedure, the anesthesia delivery was discontinued and oxygen was delivered until extubation. Atipamezole (Atipam, DECHRA Veterinary Products SAS, Suresnes, France) at 75 μg/kgBW was administered intramuscularly in case of prolonged recovery.

### RNA Extraction and RT-PCR

Total RNA was extracted from colonic tissue using TRIzol® reagent according to the manufacturer's instructions (Life Technologies, Cergy-Pontoise, France) and its concentration was measured by spectrophotometry at 260 nm.

Total RNA was reverse-transcribed into cDNA using Superscript IV Reverse Transcriptase (Life Technologies, Cergy-Pontoise, France). Complementary DNA solution (2.5 μl), was added to a mixture containing 10 μl Takyon^TM^ MasterMix (Eurogentec, Angers, France), 2 μl of each primer (2.5 mM) (sense and antisense), and 3.5 μl of ultrapure water. Real-time PCR was conducted on an CFX96 Connect system (Bio-Rad, Hercules, CA, USA). Sequences of RNA were obtained at the nucleotide database of NCBI. Primers were designed using Primer 3 input program (Palo Alto CA, USA) and presented in Table 2. The expression of GAPDH was used as reference value. Gene expression was calculated by the 2^−ΔΔ*Ct*^ method (29).

**Table 2:** Sense and antisense primers sequences.

**Genes**	**Primers sense**	**Primers antisense**
Syndecan	5′-ATTTCCGTGTTGCCAAGCTT-3′	5′-AGGCAAGTACAAGAGGTCCC-3′
Claudin 1	5′-CGGACCTATCTTTGGCGTTG-3′	5′-ACACATTCTCAGGCAGCTCT-3′
Claudin 2	5′-GGTGGGTGGAGTCTTCTTCA-3′	5′-CCAGCTACCAGGGAGAACAA-3′
Occludin	5′-TGGCGTACTCTTCCAATGGT-3′	5′-CCGTCGTGTAGTCTGTCTCA-3′
ZO-1	5′-CTAAACCTGGGGCTGTCTCA-3′	5′-AGGTAGGACGCCATCAGATG-3′
JAM	5′-GCAGCCCACTTTCTTCTGTC-3′	5′-AAAGCTCCTGTTTGGGGTTT-3′
MLCK	5′-GGCCTCTCTGACCTCAAAGT-3′	5′-TAAGTGCCTGTGTCCTCTGG-3′
TLR4	5′-CTCTCCTGGAAGGACTGTGC-3′	5′-CCGTTGCCATCTGAGATTTT-3′
TGFβ	5′-TCAAGAAAAGTCCGCACAGC-3′	5′-GCGCCAGGAATCATTGCTAT-3′
IL-10	5′-CGACCCAGACATCAAGAACC-3′	5′-CACAGGGAAGAAATCGGTGA-3′
NFκB	5′-CATCTACGACAGCAAAGCCC-3′	5′-AATCCCCAAATCCTCCCCAG-3′
TNFα	5′-CTTCTCGAACCCCAAGTGAC-3′	5′-ACCCATCTGACGCCACTATC-3′
GAPDH	5′-ACAGTCAAGGCTGAGAACGG-3′	5′-CCACAACATACTCAGCACCAGC-3′

### Microbiota Analysis

Every fortnight, dogs that were housed in pairs, were separated during the day in order to identify the stools. Fecal samples were collected immediately after spontaneous defecation and frozen in liquid nitrogen and then stored at −80°C until use. Bacterial DNA extraction from fecal samples was performed using a commercially available kit (PowerSoil® DNA isolation kit, Mo Bio Laboratories Inc., Quiagen, Carlsbad, CA, USA) according to the manufacturer's instructions.

PCR primers 515/806 (with a specific barcode for each animal) of 16S rRNA gene V4 variable region were used in a 30-cycles PCR using the master mixing kit HotStarTaq Plus (Qiagen, USA) under the following conditions: 94°C for 3 min, followed by 30 cycles of 94°C for 30 s, 53°C for 40 s, and 72°C for 1 min, after which a final elongation step at 72°C for 5 min was performed. After amplification, PCR products were checked in 2% agarose gel to determine the success of amplification. Multiple samples were pooled together in equal proportions based on their DNA concentrations. Pooled samples were purified using calibrated Ampure XP beads. Then the pooled and purified PCR product was used to prepare Illumina DNA library. Sequencing was performed at MR DNA (www.mrdnalab.com, Shallowater, TX, USA) on a MiSeq following the manufacturer's guidelines. Sequence data were processed using MR DNA analysis pipeline (MR DNA, Shallowater, TX, USA). In summary, sequences joined, depleted of barcodes, <150 bp and with ambiguous base calls were removed. Sequences were denoised, OTUs (Operational taxonomic units) generated and chimeras removed. OTUs were defined by clustering at 3% divergence. Final OTUs were taxonomically classified using BLASTn against a curated database derived from RDPII and NCBI (www.ncbi.nlm.nih.gov, http://rdp.cme.msu.edu). Raw sequences were submitted to NCBI GenBank database under BioProject number PRJNA639131.

Alpha diversity was evaluated with Chao1 (richness), Shannon diversity, and observed OTUs. Beta-diversity metric was estimated by unweighted and weighted phylogening-based UniFrac distances and visualized using Principle Coordinate Analysis (PCoA) plots.

### Statistical Analysis

Results were expressed as mean ± SEM or median (range). Results were analyzed according to their normality. *T*-test, Mann-Whitney or Wilcoxon test were used to analyze plasma parameters in the unfed state. One-way ANOVA for repeated measures was used to analyze the L:S ratio followed by a multiple comparison using Tukey. The ANOSIM (Analysis of Similarity) test within PRIMER 7 software package (PRIMER-E Ltd., Luton, UK) was used to analyze significant differences in microbial communities between groups and times. A two-ways ANOVA for repeated measures was used to analyze BW, parameters during feed-challenge test and microbiota. *P*-values were adjusted for multiple comparisons using Tukey and Benjamini and Hochberg False discovery rate and significance was set at *q* < 0.05. If residuals were not normally distributed, quantitative variables were rank-transformed if necessary. The statistical analysis was completed using Graphpad Prism 8.3.1® (San Diego, CA).

## Results

Dogs remained healthy over the duration of the study. They ate their daily ration fully as supported by weight maintenance or weight gain.

**Figure 2:**
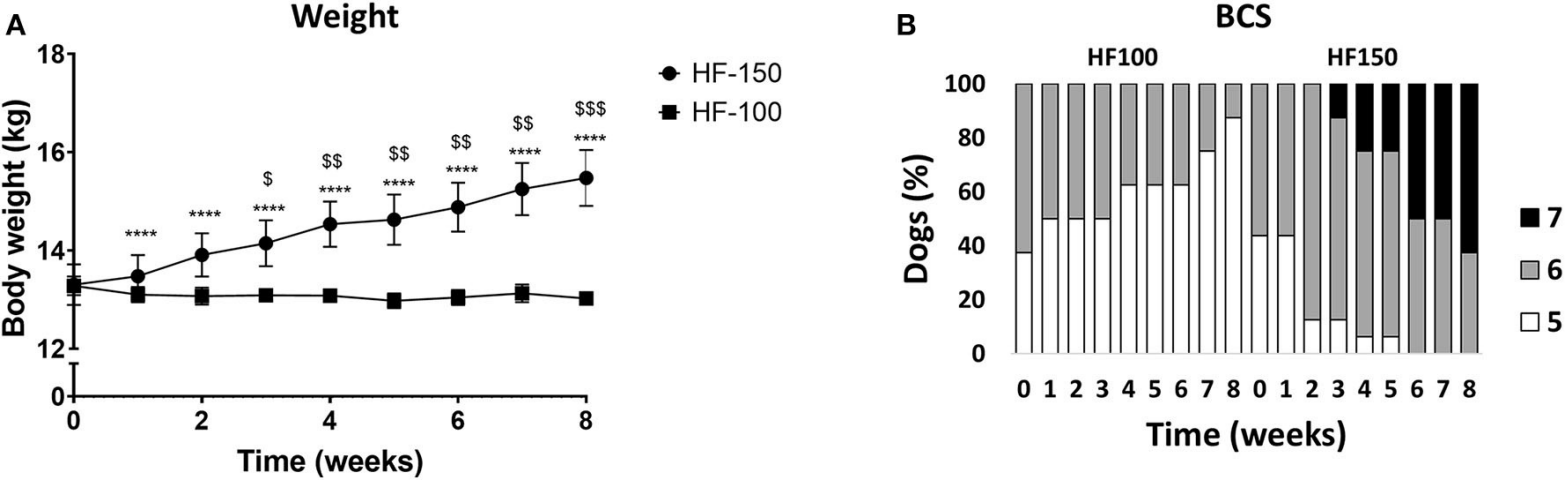
**(A)** Body weight and **(B)** Body condition score (BCS) in dogs fed the high-fat diet at maintenance (HF-100; *n* = 8) and at 150% maintenance (HF-150; *n* = 16) during 8 weeks. Data are mean ± SEM. ****P < 0.0001 for the HF-150 diet vs. week 0, ^$^*P* < 0.05, ^$^*P* < 0.01, ^$$$^*P* < 0.001 HF-150 vs. HF-100 at the same week.

After 8 weeks of diet, BW and BCS were significantly higher in HF-150 group compared to the HF-100 group. In HF-150 group, the mean BW increased by 17.4 ± 6.5 % over the 8-week intervention and this was associated with an increase of BCS (week 8: median BCS 7; range 6–7 vs. week 0: median BCS 6; range 5–6) (Figures 2A,B).

### Effect of Dietary Regime on Microbiota

All microbiota data are compiled in Supplementary Table 1.

**Figure 3:**
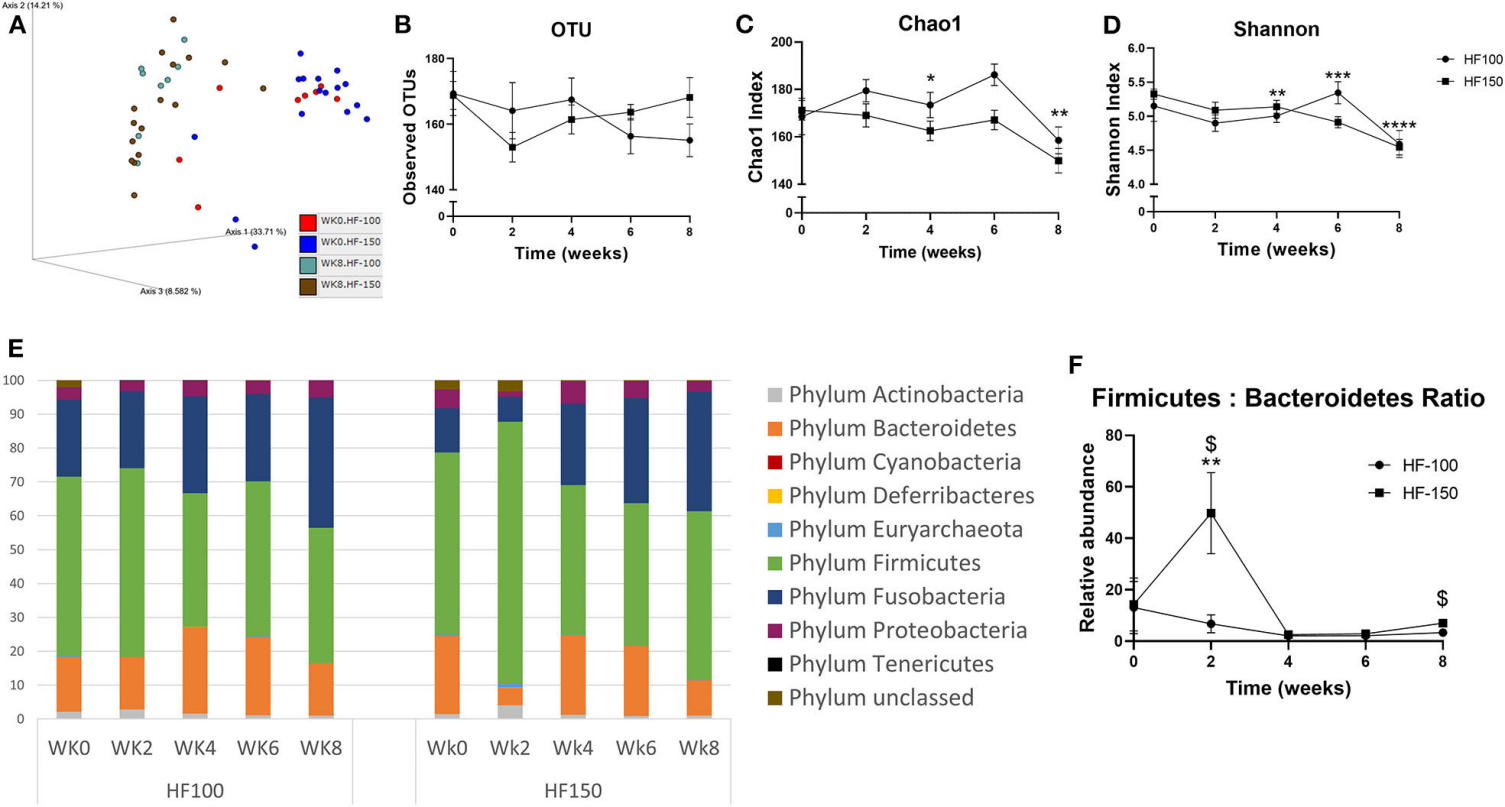
**(A)** The β-diversity, the indices of **(B)** OTUs, **(C)** Chao1 and **(D)** Shannon, **(E)** relative abundance of bacterial on phyla level and **(F)** Firmicutes/Bacteroidetes ratio found in fecal samples of dogs fed the high-fat diet at maintenance (HF-100; *n* = 8) and at 150% maintenance (HF-150; *n* = 16) for 8 weeks. Data are mean ± SEM. *P < *0.05*, **P < *0.01*, ***P < *0.001*, ****P < *0.0001* during the HF-150 diet vs. week 0, ^$^*P* < 0.05 HF-150 vs. HF-100 at weeks 2 and 8.

Analysis of β-diversity revealed significantly distinct clustering during the diet period compared to week 0 for both groups (Figure 3A). PCoA plot based on weighted UniFrac distance metric showed that week 8 clustered separately from the cluster of week 0 in HF-150 group (_weighted_ANOSIM; *p* = *0.001, R* = *0.706*) and in HF-100 group (_weighted_ANOSIM; *p* = *0.001, R* = *0.54*).

At the α-diversity level, observed OTUs were not significantly different at week 8 compared to week 0 in both groups (Figure 3B). In contrast to the HF-100 group where no significant changes in α-diversity richness and evenness were observed over time, the HF-150 group had significantly lower α-diversity richness and evenness, as indicated by Shannon and Chao1 indexes, respectively. Indeed, in the HF-150 group, Shannon index was significantly lower from the fourth week of intervention (*p* = *0.03*) to week 8 (*p* = *0.0001*) and Chao1 index was significantly lower at week 4 (*p* = *0.04*) and week 8 (*p* = *0.004*) compared to week 0 (Figures 3C,D).

The more represented phyla were Firmicutes, Bacteroidetes, Fusobacteria, Proteobacteria, and Actinobacteria. The phyla Euryarchaeota, Deferribacteres, Tenericutes were present only in a subset of dogs. No significant changes in the relative abundance of the predominant phyla were observed in the HF-100 group. On the other hand, many changes were observed in the HF-150 group. The relative abundance of phyla Firmicutes (*q* = *0.0002*; wk2 vs. wk0 *p* = *0.0002*) and Actinobacteria (*q* = *0.0002*; wk2 vs. wk0 *p* = *0.004*) were only increased at week 2 in HF-150 group (Figure 3E). In this group, the relative abundance of Fusobacteria was initially significantly decreased at week 2 (*q* = *0.0002*; wk2 vs. wk0 *p* = *0.009*) and then increased at weeks 4, 6, and 8 (*q* = *0.0002*; wk4 vs. wk0 *p* = *0.0003*; wk6 vs. wk0 *p* = *0.0001*; wk8 vs. wk0 *p* = *0.0001*), whilst Bacteroidetes abundance (*q* = *0.0002*; HF-150 wk2 vs. wk0 *p* = *0.0002*; wk8 vs. wk0 *p* = *0.003*) and Proteobacteria abundance (*q* = *0.008*; HF-150 wk2 vs. wk0 *p* = *0.0007*; wk8 vs. wk0 *p* = *0.04*) were significantly lower at week 2 and week 8 compared to week 0 (Figure 3E). In HF-150 group, the Firmicutes/Bacteroidetes ratio was greater at week 2 compared to week 0 (*p* = *0.01*) (Figure 3F), and was higher in HF-150 group compared to HF-100 group at week 2 (*p* = *0.02*) and week 8 (*p* = *0.04*) (Figure 3F).

**Figure 4:**
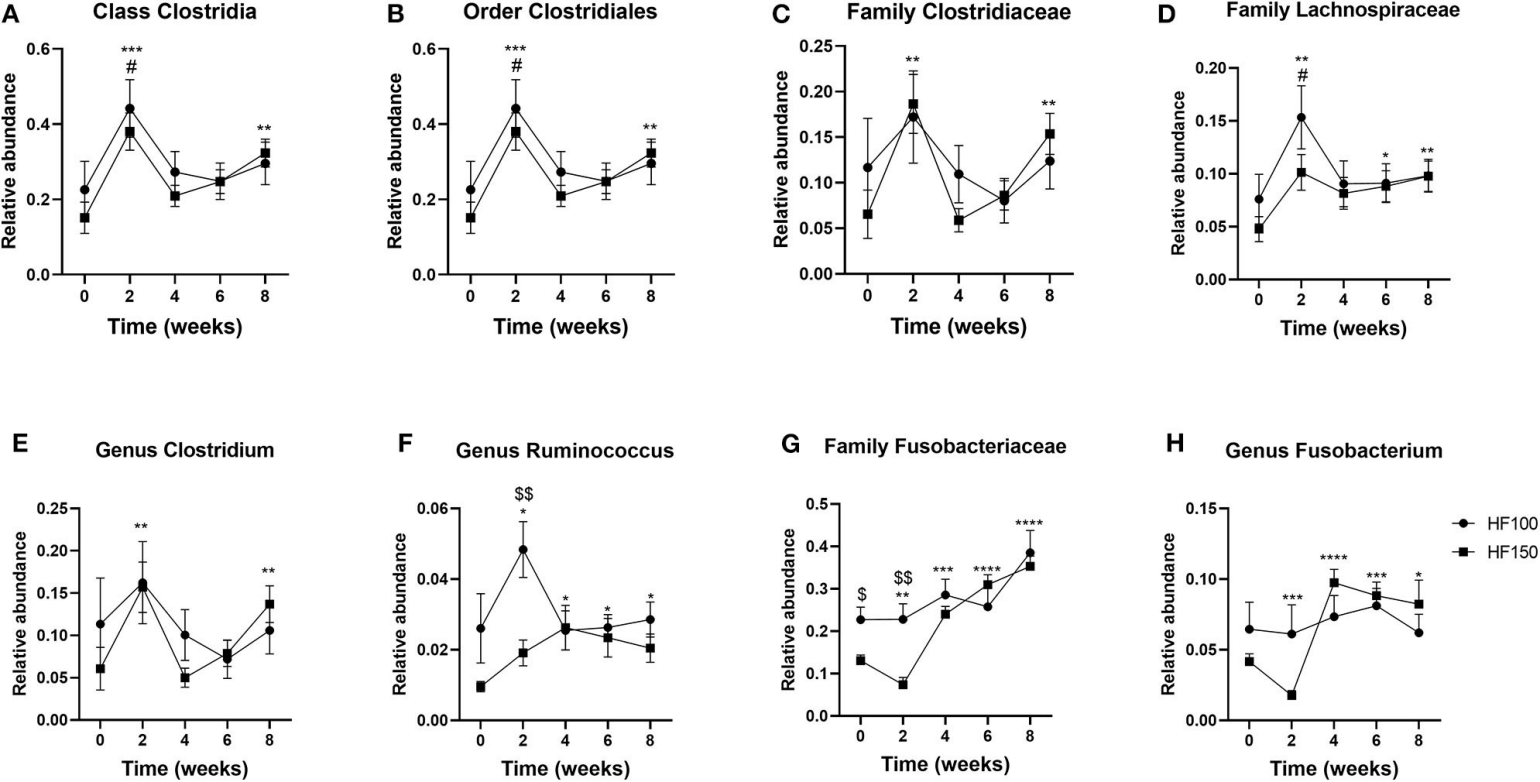
Relative abundance of the fecal bacterial groups belonging to the phyla Firmicutes [**(A)** Clostridia, **(B)** Clostridiales, **(C)** Clostridiaceae, **(D)** Lachnospiraceae, **(E)** Clostridium, and **(F)** Ruminococcus] and Fusobacteria [**(G)** Fusobacteriaceae and **(H)** Fusobacterium] in dogs fed the high-fat diet at maintenance (HF-100; *n* = 8) and at 150% maintenance (HF-150; *n* = 16) for 8 weeks. Data are mean ± SEM. *P < 0.05, **P < 0.01, ***P < 0.001, ****P < 0.0001 during the HF-150 diet vs. week 0, ^#^*P* < 0.05 at week 2 vs. week 0 for HF-100 group, ^$^*P* < 0.05, ^$$^*P* < 0.01 HF-150 vs. HF-100 at weeks 0 and 2.

Within the phylum Firmicutes, the relative abundance of Clostridia class was significantly greater in HF-150 group at week 8 compared to week 0 (*q* = *0.0003*; wk8 vs. wk0 *p* = *0.003*), which was driven by only the order Clostridiales (*q* = *0.0004*; wk8 vs. wk0 *p* = *0.003*), and the families Clostridiaceae (*q* = *0.007*; wk8 vs. wk0 *p* = *0.002*), and Lachnospiraceae (*q* = *0.02*; wk8 vs. wk0 *p* = *0.008*) (Figures 4A–D). At the genus level, *Clostridium* (*q* = *0.02*; wk8 vs. wk0 *p* = *0.004*), and *Ruminococcus* (wk8 vs. wk0 *p* = *0.02*) were significantly more abundant in the HF-150 group at week 8 compared to week 0 (Figures 4E,F). The only family present in the Fusobacteria phylum was Fusobacteriaceae. In the HF-150 group, the relative abundance of Fusobacteriaceae decreased at week 2 and then increased in the following weeks (*q* = *0.001*; wk2 *p* = *0.01*, wk4 *p* = *0.0003*, wk6 *p* < *0.0001*, wk8 *p* < *0.0001* vs. wk0) driven in particular by the genus Fusobacterium (*q* = *0.01*; wk2 *p* = *0.0007*, wk4 *p* < *0.0001*, wk6 *p* = *0.0009*, wk8 *p* = *0.03* vs. wk0) (Figures 4G,H).

**Figure 5:**
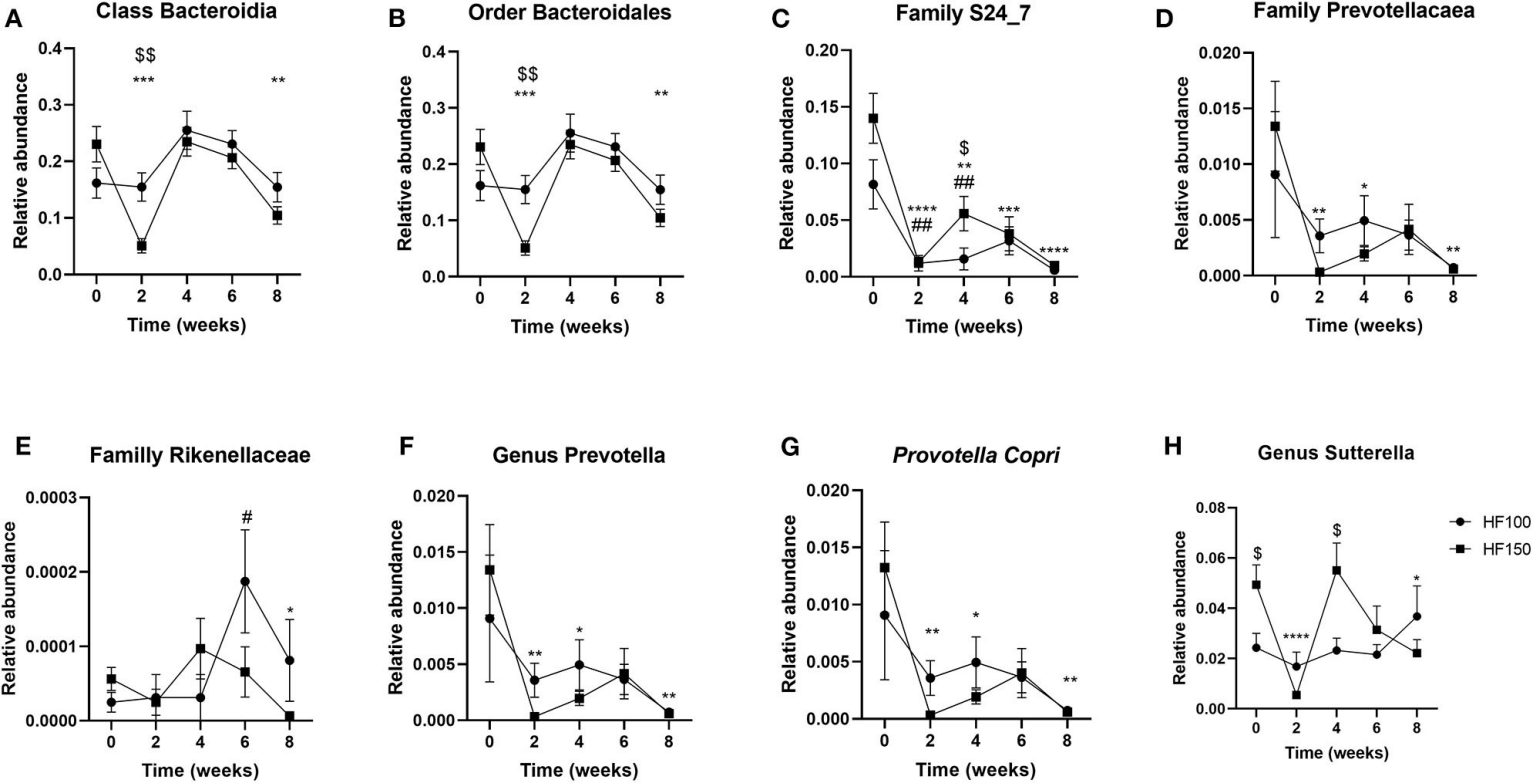
Relative abundance of the fecal bacterial groups belonging to the phylum Bacteroidetes [**(A)** Bacteroidia, **(B)** Bacteroidales, **(C)** S24_7, **(D)** Prevotellacaea, **(E)** Rikenellaceae, **(F)** Prevotella, and **(G)** Prevotella Copri] and Proteobacteria [**(H)** Sutterella] in dogs fed the high-fat diet at maintenance (HF-100; *n* = 8) and at 150% maintenance (HF-150; *n* = 16) for 8 weeks. Data are mean ± SEM. *P < 0.05, **P < 0.01, ***P < 0.001, ****P < 0.0001 during the HF-150 diet vs. week 0, ^#^*P* < 0.05, ^*##*^*P* < 0.01 at week 2, 4, and 6 vs. week 0 for HF-100 group, ^$^*P* < 0.05, ^$$^*P* < 0.01 HF-150 vs. HF-100 at weeks 0, 2, and 4.

The phylum Bacteroidetes was composed in its totality by the class Bacteroidia, which Bacteroidales was the only order identified (Figures 5A,B). In the HF-150 group, the relative abundance of Bacteroidales decreased at weeks 2 and 8 (*q* = *0.0004*; wk2 *p* = *0.0002*, wk8 *p* = *0.003* vs. wk0) (Figure 5B). The families Prevotellaceae (*q* = *0.03*), Rikenellaceae (*q* = *0.04*) and S24_7 (*q* = *0.001*) were less abundant in HF-150 group over study time compared to week 0 (Figures 5C–E). At species level, the relative abundance of *Prevotella copri*, the only species detected within the genus *Prevotella*, decreased in HF-150 group at weeks 2, 4, and 8 (*q* = *0.04; wk2 p* = *0.005, wk4 p* = *0.01, wk8 p* = *0.006* vs. wk0) (Figures 5F,G).

Belonging to the phylum Proteobacteria, the genus *Sutterella*, the only one present in the family Alcaligenaceae, was significantly less abundant in the HF-150 group at weeks 2 and 8 compared to week 0 (*q* = *0.03*; wk2 *p* < *0.0001*, wk8 *p* = *0.02* vs. wk0) (Figure 5H).

### Effect of Dietary Regime on Digestibility

At the end of the study, there was only subtle differences in apparent total tract digestibility. The HFD did not lead to a significant modification of the digestibility of organic matter (HF-100 wk8 = 91.2 ± 0.6% vs. wk0 = 90.9 ± 0.4% *p* = *0.70*; HF-150 wk8 = 94.2 ± 0.3% vs. wk0 = 93.4 ± 0.4% *p* = *0.11*) and crude protein (HF-100 wk8 = 90.7 ± 0.5% vs. wk0 = 90.3 ± 0.5%; *p* = *0.62*; HF-150 wk8 = 93.8 ± 0.4% vs. wk0 = 93.3 ± 0.4% *p* = *0.38*) in either group. Fat digestibility was significantly higher in the HF-100 group at week 8 compared to week 0 (HF-100 wk8 = 98.3 ± 0.2% vs. wk0 = 97.9 ± 0.1% *p* = *0.01*), but this difference could not be considered as physiologically significant. No difference was observed in the HF-150 group (HF-150 wk8 = 98.8 ± 0.1% vs. wk0 = 98.7 ± 0.1% *p* = *0.24*) (Supplementary Table 2).

### Effect of Dietary Regime on Intestinal Barrier and Inflammation

**Figure 6:**
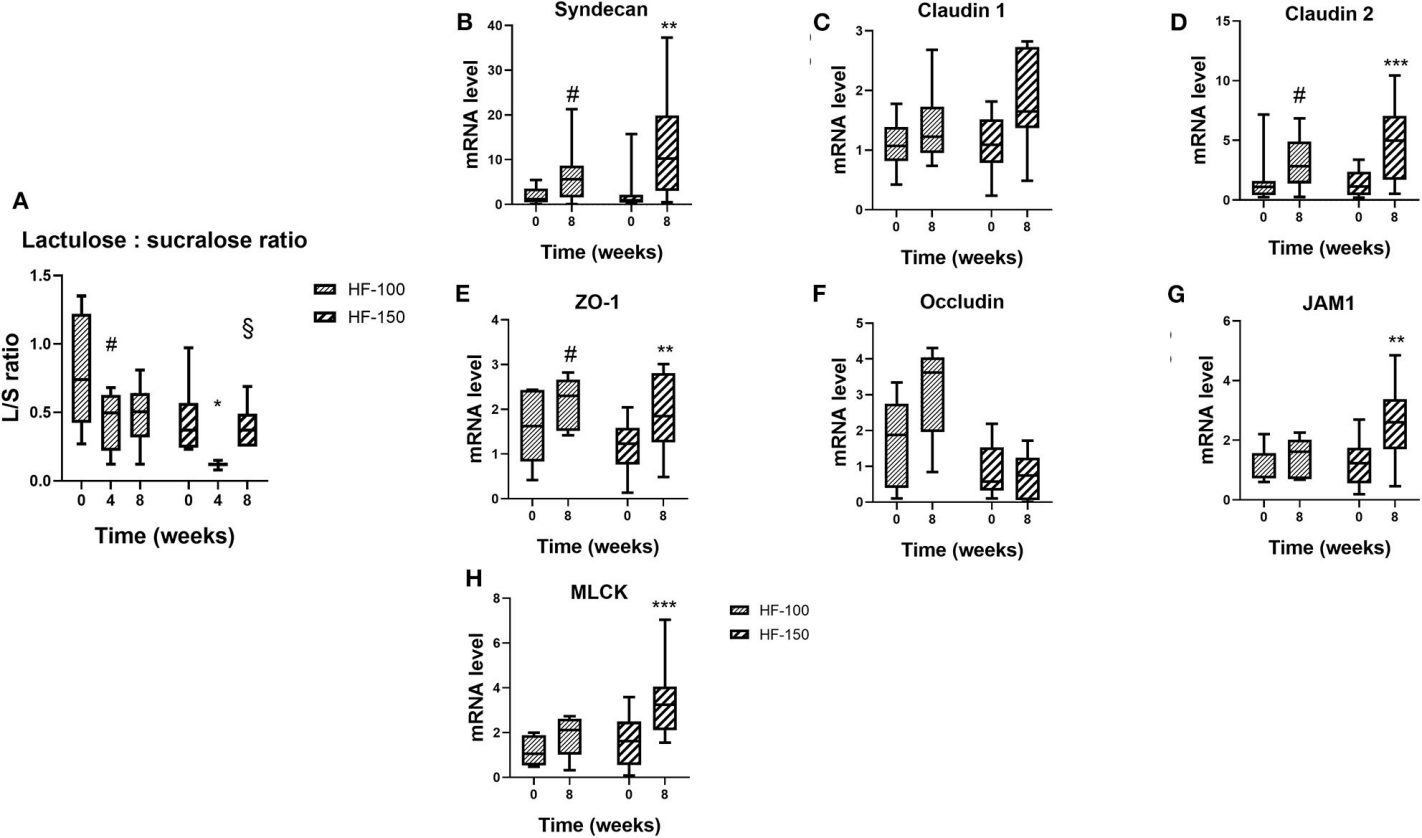
**(A)** Lactulose:sucralose ratio in urine after an oral administration and **(B)** Syndecan, **(C)** Claudin-2, **(D)** ZO-1, **(E)** Junctional adhesion molecule 1 (JAM1), **(F)** Myosin light-chain kinase (MLCK), **(G)** Claudin-1, and (H) Occludin mRNA levels in dogs fed the high-fat diet at maintenance (HF-100; *n* = 8) and at 150% maintenance (HF-150; *n* = 16) for 8 weeks. Data are median (range min-max). *P < 0.05, **P < 0.01, ***P < 0.001 at weeks 4 and 8 vs. week 0 for HF-150 group, ^§^*P* < 0.05 at week 8 vs. week 4 for HF-150 group, ^#^*P* < 0.05 at weeks 4 and 8 vs. week 0 for HF-100 group.

The urinary L:Sratio in dogs is shown in Figure 6A. The urinary L:S ratio was significantly lower at week 4 compared to week 0 for both groups, indicating a higher colonic permeability (HF-100 *p* = *0.03*; HF-150 *p* = *0.02*). In the HF-150 group, the L:S ratio was significantly higher at week 8 compared to week 4 (*p* = *0.004*). The L:S ratio was similar at week 8 compared to week 0 for both groups (HF-100 *p* = *0.10*; HF-150 *p* = *0.42*).

In both groups at week 8, mRNA expressions of syndecan (HF-100 *p* = *0.04*, HF-150 *p* = *0.001*), claudin 2 (HF-100 *p* = *0.04*, HF-150 *p* = *0.0002*) and ZO1 (HF-100 *p* = *0.03*, HF-150 *p* = *0.004*) were significantly higher compared to week 0 (Figures 6B–D). JAM1 and MLCK mRNA expression were significantly higher at week 8 compared to week 0 only for the HF-150 group (respectively, *p* = *0.002*; *p* = *0.0008*) (Figures 6E,F). No significant differences in the expressions of claudin 1 (HF-100 *p* = *0.45*, HF-150 *p* = *0.07*) or occludin (HF-100 *p* = *0.17*, HF-150 *p* = *0.47*) were found between weeks 8 and 0 in both groups (Figures 6G,H). At week 8, we did not observe significant differences in IL-10 (*p* = *0.41*), NFKB (*p* = *0.69*), and TNFα (*p* = *0.83*) mRNA expression levels in colonic biopsies between HF-100 and HF-150 groups. The HF-150 group had decreased expressions of TLR4 (*p* = *0.01*) and TGFβ (*p* = *0.001*) mRNA compared to week 0. The TLR4 mRNA expression was significant lower in the HF-150 group compared to the HF-100 group (*p* = *0.03*) while TGFβ mRNA expression was found to be unchanged (*p* = *0.23*).

Plasma inflammatory markers were measured and are described as median (range). Plasma CRP [HF-150: 1.8 (0.8–12.5) mg/ml vs. HF-100: 0.8 (0.4–1.9) mg/ml; *p* = *0.18*], SAA [HF-150: 13.5 (13.4–15.0) μg/ml vs. HF-100: 13.5 (13.4–15.4) μg/ml; *p* = *0.94*], and haptoglobin [HF-150: 1.78 (1.08–4.42) mg/ml vs. HF-100: 1.77 (1.13–10.17) mg/ml; *p* = *0.83*] concentrations were not significantly different at week 8 in both groups. Oxidative stress markers were also measured. Plasma SOD [HF-150: 0.61 (0.30–0.89) U/ml vs. HF-100: 0.53 (0.12–0.78) U/ml; *p* = *0.35*], GPx [HF-150: 57 (36–108) U/g Hg vs. HF-100: 87 (35–132) U/g Hg; *p* = *0.37*], and TAS [HF-150: 0.78 (0.45–1.20) mmol/ml vs. HF-100: 0.77 (0.25–1.12) mmol/ml; *p* = *0.63*] concentrations were not significantly different at week 8 in both groups. Plasma LPS [HF-150: 0.15 (0.08–0.51) EU/ml vs. HF-100: 0.21 (0.09–0.31) EU/ml; *p* = *0.54*] and LBP concentrations [HF-150: 11.2 (3.5–31.8) ng/ml vs. HF-100: 15.3 (6.1–55.7) ng/ml; *p* = *0.54*] was similar in HF-100 and HF-150 groups at 8 weeks.

### Effect of Dietary Regime on Metabolic Parameters

In the HF-150 group, plasma leptin concentration was higher [wk8: 4.5 (1.0–9.0) ng/ml vs. wk0: 2.0 (1.0–4.0) ng/ml; *p* = 0005], plasma adiponectin concentration was lower [wk8: 69.9 (15.8–283.5) ng/ml vs. wk0: 133.6 (40.7–287.0) ng/ml; *p* = 0.02] and plasma ghrelin concentration was similar [wk8: 0.49 (0.15–1.59) ng/ml vs. wk0: 0.56 (0.07–1.73) ng/ml; *p* = 0.08] at week 8 compared to week 0. In the HF-100 group, leptinemia, adiponectinemia, and ghrelinemia were not significantly different at week 8 compared to week 0. The variation in leptinemia, between weeks 0 and 8 (Δwk8/wk0), was higher in HF-150 group compared to HF-100 group [Δwk8/wk0: HF-150: 2 (0–6) vs. HF-100: 0 (−2–+3); *p* = 0.036], while it was lower in adiponectinemia (Δwk8/wk0: HF-150:−65 (−196–+75) vs. HF-100: 32 (−7–+69)), and not statistically different in ghrelinemia (*p* = 0.904).

**Figure 7:**
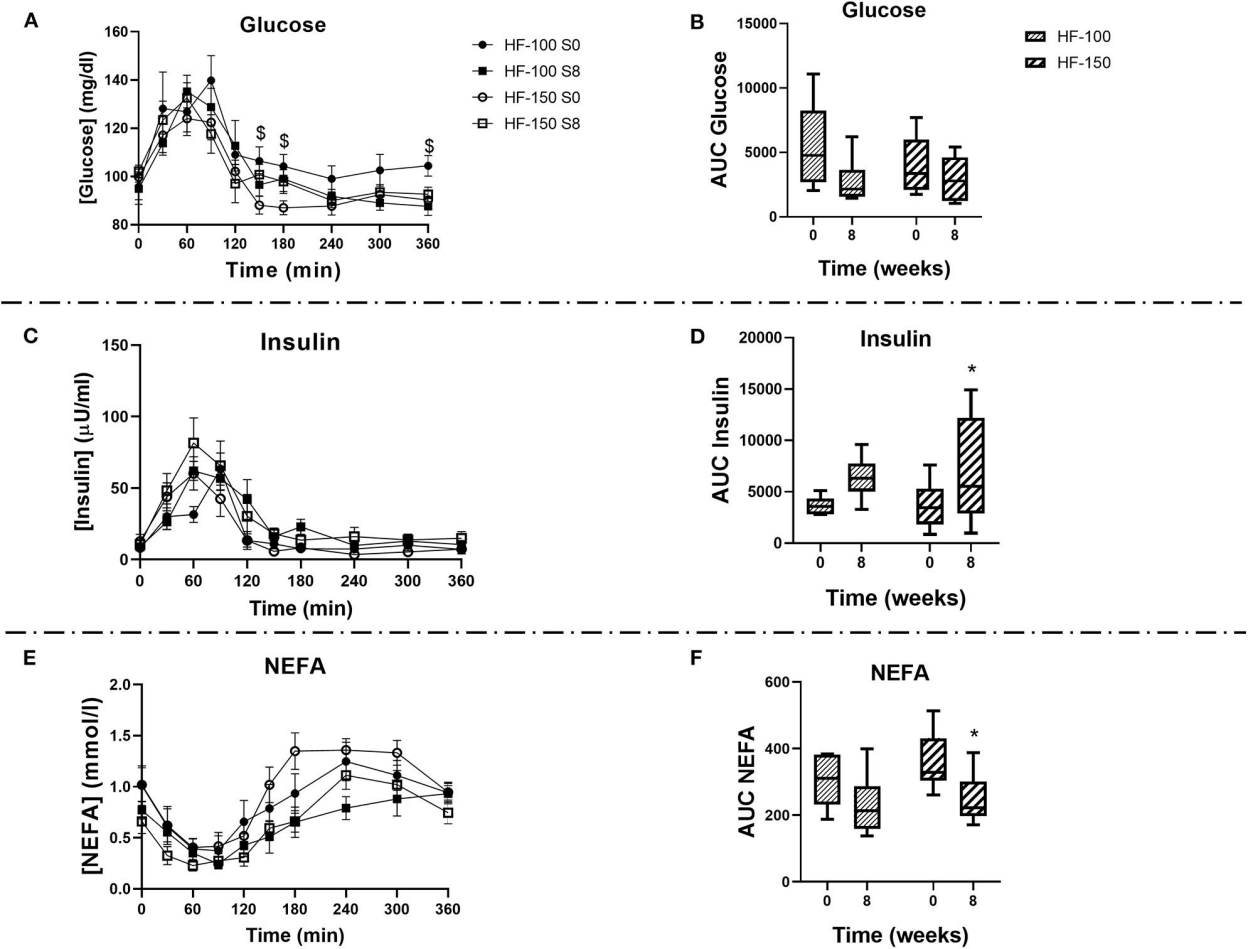
**(A)** Plasma glucose, **(C)** Plasma insulin **(E)**, and Plasma NEFA concentrations, and the respective areas under the curves **(B,D,F)** during a feed-challenge test in dogs fed the high-fat diet at maintenance (HF-100; *n* = 8) or at 150% maintenance (HF-150; *n* = 8) for 8 weeks. Data are mean ± SEM or median (range min-max). ^$^*P* < *0.05* HF-150 vs. HF-100 for the same timepoint at week 0. *P < *0.05* at week 8 vs. week 0 for HF-150 group.

Basal glucose (HF-100: *p* = *0.71*, HF-150: *p* = *0.37*) and insulin (HF-100: *p* = *0.90*; HF-150: *p* = *0.43*) concentrations were not significantly changed at week 8 compared to week 0 in both groups (Table 3). In addition, no changes in plasma glucose response following the feed-challenge test in both groups were observed (Figures 7A,B). The insulin response, which has been estimated by the AUC after feed-challenge, was not modified in HF-100 group (AUC wk0: 3585 ± 422 AU; AUC wk8: 6386 ± 687 AU; *p* = *0.14*). In the HF-150 group, the insulin response was higher at week 8 (AUC = 3678 ± 775) compared to baseline (AUC = 7165 ± 1889; *p* = *0.04*), which reflects a lower insulin sensitivity (Figures 7C,D). Comparing week 8 to week 0, TG, total cholesterol and HDL-C concentrations were not different either in basal state or postprandially in both groups (Supplementary Figure 1). The response in NEFA to the feed-challenge was lower at week 8 (AUC: 249 ± 28 AU) compared to week 0 (AUC: 358 ± 32 AU; *p* = *0.02*) in the HF-150 group despite being not significantly different in basal state (*p* = *0.07*) (Table 3, Figures 7E,F).

**Table 3:** Basal concentrations of glucose, insulin, TG, total cholesterol, HDL-C, NEFA, GIP, and NPY at week 0 and at week 8 for HF-100 and HF-150 groups.

	**HF-100 (*****n*** **=** **8)**		**HF-150 (*****n*** **=** **16)**	
	**Wk0**	**Wk8**	**Wk0**	**Wk8**
Glucose (mg/dl)	95.8 ± 7.3	94.9 ± 4.5	99.8 ± 2.1	102.0 ± 2.5
Insulin (μU/ml)	7.8 ± 0.8	8.9 ± 2.1	8.6 ± 1.3	10.6 ± 2.3
TG (mg/dl)	119.2 ± 41.7	126.0 ± 43.0	66.5 ± 12.4	88.9 ± 32.4
Total cholesterol	215.0 ± 13.5	189.1 ± 10.2	195.8 ± 13.9	198.5 ± 5.1
(mg/dl)				
HDL-C (mg/dl)	108.0 ± 19.8	88.6 ± 13.7	82.3 ± 10.0	87.8 ± 9.9
NEFA (mmol/l)	1.0 ± 0.2	0.8 ± 0.1	1.0 ± 0.2	0.7 ± 0.2
GIP (ng/ml)	1.4 ± 0.8	1.6 ± 0.9	1.3 ± 0.6	2.3 ± 1.1
NPY (ng/ml)	1.1 ± 0.4	1.2 ± 0.4	1.5 ± 0.4	1.3 ± 0.4

*Data are mean ± SEM*.

## Discussion

The overarching aim of this longitudinal study was to evaluate the effects of a HFD administered at two energy levels on the fecal microbiota, the colonic barrier, and the metabolic and inflammatory status in dogs. The HFD fed at maintenance energy requirements induced alteration in the relative abundance of fecal bacteria at every taxonomic level, from phylum to species, a transient increase in colonic permeability, while there was no disturbance of metabolic and inflammatory status. The same HFD fed at 150% maintenance requirements decreased fecal bacterial diversity and altered the relative abundance of bacteria. It also induced a transient increase in colonic permeability, a lower insulin sensitivity and a disruption in adipokine levels.

As expected, feeding dogs at 150% of their maintenance energy requirements led to weight gain and an increase in BCS. It has been described that basal plasma leptin concentration increases while adiponectin concentration decreases when the fat mass increases (20, 30). In our study, increased leptin concentration and the decreased adiponectin concentration measured in HF-150 dogs were consistent with the expansion of the adipose tissue.

A decrease in α-diversity was found only at week 8 in dogs fed at 150% maintenance. Similar results have been described in obese companion dogs as well as in obese colony dogs induced by an *ad libitum* maintenance diet (16% fat, 25% protein, 3868 kcal/kg) (20, 21). The increase in energy intake and not the fat intake could lower the diversity of the microbiota which could be an early marker of obesity.

The dynamics of the intestinal microbiota in the context of diet modification (changes in macronutrient availability and energy level) was evaluated in our study. We identified significant changes in the relative abundance of several bacterial phyla as early as on the second week after the HF-diet had been introduced, exclusively in HF-150 dogs. Among these changes, the relative abundance of Actinobacteria and Firmicutes increased, while the relative abundance of Bacteroidetes, Fusobacteria, and Proteobacteria decreased. Despite identical diet conditions in the following weeks, only the relative abundance of Bacteroidetes and Proteobacteria remained lower at week 8 compared to the beginning, while the relative abundance of Actinobacteria and Firmicutes returned to basal levels and the relative abundance of Fusobacteria increased. In rats fed with a HF-diet (45% fat vs. Control, 13% fat), the microbiota exhibited resilience after the initial perturbations caused by diet change (31). The resilience, which is notably driven by the initial microbial diversity, means that the amount of perturbation (e.g., a diet change) imposed on the microbial ecosystem is tolerated so that its trajectory does not definitely move toward a different equilibrium state (32).

A lower relative abundance of Bacteroidetes and a higher ratio of Firmicutes/Bacteroidetes have been found in HF-150 dogs, and this could be linked to the BW gain. Although several studies have tried to describe the microbiota in obese dogs, they did not succeed to highlight a unique bacterial profile that would be associated with the obese state. When comparing the phyla composition in obese dogs and lean ones, one study showed no significant difference in relative abundance at the phylum level (21). In another study, a higher relative abundance of Actinobacteria has been described (12), while in a third one a lower relative abundance of Firmicutes and a higher relative abundance of Proteobacteria was reported (20). In obese mice (18) and humans (19, 33), an increased Firmicutes/Bacteroidetes ratio has been reported, and the relative abundance of these phyla has been suggested as an indicator or factor for obesity (34). Moreover it has been shown that the Firmicutes/Bacteroidetes ratio decreased along a dieting and subsequent weight-losing period in obese humans (19). In obese compared to lean twins, Firmicutes were dominant in the microbiota and the microbiome was enriched with genes known to be associated with nutrient transporters (35). A possible explanation of the effect of microbiota on the development of obesity might be that the microbiota of obese individuals have an increased capacity to ferment non-digestible carbohydrates and produce SCFA, which will be subsequently absorbed, metabolized to more complex lipids in the liver, and then stored in adipose tissue (22, 23, 36). Thus, the higher Firmicutes/Bacteroidetes ratio we observed in the overweight dogs might have been a factor of weight gain promotion.

Among the Firmicutes, we observed an increase in the Clostridiales order in HF-150 dogs at week 8 compared to week 0, driven by increases in three Clostridiales families, Clostridiaceae, Lachnospiraceae and Peptostreptococcaceae. At the genus level, *Ruminococcus* (Lachnospiraceae) and *Clostridium* (Clostridiaceae) were increased. A higher relative abundance of Ruminococcus has been reported in rats fed a HFD(45% energy from fat) compared to ones consuming a control diet (10% energy from fat), and Ruminococcus abundance was positively correlated with amount of fecal deoxycholic acid (DCA) (37). A positive correlation between cecal bile acid concentration and specific bacteria of the order Clostridiales in mice fed a HFD has been shown (38). An extensive conversion of cholic acid into DCA in the cecal contents associated with an increase in the class of Clostridia has been shown in rats fed a diet supplemented with cholic acid (39). In dogs whose diet was shifted from commercial dry food (16.3% fat) to one based on minced beef (33.1% fat) and vice versa, changes in the fecal bile acid concentrations and profiles were observed (40). So, in HF-150 dogs, the higher lipid intake could have contributed to an increase in the gut lumen bile acid content and the relative abundance of genera belonging to the Clostridiales order such as Ruminococcus and Clostridium.

Within the phylum Bacteroidetes, the relative abundance of family S24-7, Prevotellaceae and Rikenellaceae were lower in HF-150 dogs at week 8 compared to week 0. Within Prevotellaceae, the relative abundance of *P. copri*, the only species belonging to the Prevotella genus that was found, was also lower in HF-150 dogs at week 8 compared to week 0. It has been shown that the growth of *Prevotella* can be completely inhibited *in vitro* by a 20% inclusion of bile salts (41). Moreover, *Prevotella* has been found to be predominant in the rumen of sheep, where obviously bile acids are not present (42). In dogs, a previous study described a lower relative abundance of *Prevotella* in animals fed a high-fat and low-starch diet compared to animals fed a low-fat and high-starch diet independently of obesity, and *Prevotella* abundance was negatively correlated with the fecal concentration of taurocholic and taurolithocholic acids (13). Therefore, the lower relative abundance of *Prevotella* driven by *P. copri* could suggest a negative impact of dietary fat which could be due to an increased arrival of bile acids into the gut.

In our study, the relative abundance of the phylum Fusobacteria, driven by the genus Fusobacterium, was higher, and that of Proteobacteria, driven by the genus *Sutterella*, was lower in HF-150 dogs at week 8 compared to week 0. No changes in the abundance of these phyla were observed in the group fed at maintenance. In the literature, no changes in these phyla have been reported following a HFD in dogs (12). A lower abundance of Proteobacteria was observed in dogs fed a natural diet (protein/fat-based) compared to kibble diet (carbohydrate based) (43). Moreover, Fusobacteria abundance has been described to be higher in dogs fed a raw red meat diet (76% protein, 18% fat) compared to a kibble diet (30% protein, 27% fat) (44), which suggested an effect of dietary protein. On another hand, a lower Fusobacteria abundance was observed in dogs fed a “beet pulp” diet (28% protein, 21% fat, 4.5% total dietary fiber, TDF) compared to a control diet (almost same protein and fat content, 1.4% TDF) (45), which suggested an effect of dietary fiber. *Fusobacterium spp*., which are members of the Fusobacteria phyla, ferment amino acids to butyrate (46). Dogs fed at 150% maintenance have consumed 50% more protein than dogs fed at maintenance. The increase in the relative abundance of Fusobacteria and the decrease in the relative abundance of Proteobacteria in HF-150 dogs could be explained by the higher protein intake. As these dogs also consumed 50% more dietary fiber, our results might suggest a stronger effect of dietary protein.

In the current study, microbiota was assessed using 16S rRNA gene pyrosequencing. Quantitative real-time PCR and analysis of the metagenome and metabolome (in feces and plasma) would provide deeper insight into functional modifications of the microbiota. In addition, the duration of the nutritional intervention could be considered as short, and reports on the chronic effects of a HFD with or without weight gain are sparse.

In our study, colonic permeability, as determined by the L:S ratio (28), was transiently increased in both groups at week 4 followed by a restoration of a lower permeability at week 8. At the same time, the expression of colonic mucosa tight-junction proteins was altered. Despite variability in sucralose absorption in dogs (47), the L:S ratio method is the only method that allows assessment of the colonic permeability *in vivo* and in a non-invasive manner. Numerous studies have demonstrated that dietary fat induced alterations in intestinal microbiota, and related them to changes in gut permeability (26, 48–50). In rodents, obesity induced by a HFD has been associated with an increase in intestinal permeability and a decreased expression of ZO1, claudin-1 and occludin (26, 51–53) and an increased expression of Claudin-2 (52). Tight junction proteins perform different functions, either in barrier formation (decreasing paracellular permeability) or playing a role in pore formation (increasing paracellular permeability). Claudin-1, JAM-1, ZO-1, occludin, and syndecan act as barrier-forming proteins (54–57), while claudin-2 and MLCK act as pore-forming proteins (58, 59). In mice fed a HF-diet for 4 weeks, an increase in colonic permeability was observed, and was correlated to a reduction of the expression of tight-junction proteins, ZO-1 and occludin (26), and that was completely reversed by the antibiotic treatment, suggesting an important role of the microbiota in intestinal permeability (26). The changes in the microbiota observed in both groups in the present study suggest a role of gut microbiota on intestinal permeability. Only one study has shown a transient increase in intestinal permeability in mice fed a HFD (31). In our study, the higher expression of ZO-1, syndecan and JAM-1 was associated with the return of colonic permeability to initial level. The rise of the expression of these genes might indicate a compensatory mechanism to restore normal permeability.

Increased intestinal permeability can play a role in induction of metabolic disorders. In mice fed a HF-diet for 4 weeks, increased intestinal permeability facilitated transepithelial or paracellular diffusion of bacterial compounds such as LPS, which plasma concentration was notably correlated with inflammation and oxidative stress markers (26). In our study, plasma LPS concentration was similar in the unfed state in dogs fed at 100 or 150% maintenance for 8 weeks. Thus, the higher level of dietary fat did not result in a measurable low-grade endotoxemia. Despite a transient increase in intestinal permeability and significantly modified microbiota, we did not observe evidence of endotoxemia or systemic inflammation at the end of the study.

In both groups, there were no changes in plasma basal glucose, insulin, TG, cholesterol, HDL-C, NEFA, GIP, and NPY concentrations at week 8 vs. week 0. In response to the feed-challenge, TG, total cholesterol and HDL-C were similar at week 8 compared to week 0. In HF-150 dogs, the area under the curve of the insulin response to a feed challenge was higher at the end of the study. The insulin response to a feed challenge test reflects insulin sensitivity (60, 61). Thus, dogs in the HF-150 group had a lower insulin sensitivity. This finding is similar to previous work examining the link between insulin resistance and canine obesity (15, 62–64). Insulin resistance has been associated with changes in the composition of the intestinal microbiota in obese dogs compared to healthy ones (65). However, in our study, the effect of the intestinal microbiota on insulin sensitivity could not be explained by an increase in endotoxemia and inflammation as postulated from a mice study (26). Further investigation is needed to elucidate how intestinal microbiota changes can modulate the insulin sensitivity. Adipokines play an important role in the regulation of glucose homeostasis independent of food intake or BW. In humans, leptinemia and insulinemia are positively correlated to each other, regardless of the degree of adiposity of the patients (66). Leptin alters insulin signaling in murine adipocytes (67). It has been reported that insulin resistance in lipoatrophic mice has been reversed by the combination of physiological doses of adiponectin and leptin, but only partially by either adiponectin or leptin alone (68). In our study, the higher leptin concentration and reduced adiponectin concentration in dogs fed at 150% maintenance could thus have participated in the decrease in insulin sensitivity.

The dogs used in this study have common characteristics such as breed, age, and sex and were housed under the same conditions which is not representative of the whole dog population. Indeed, sex, age, breed, and kinship are likely to play a role in the composition of the intestinal microbiota (69–71). In addition, diet appears to induce variations in the microbiota that could differ between males and females (7). However, we decided to reduce these confounding factors by optimally homogenizing the canine population studied in order to optimize the statistical power of the effects of the HFD on the measured parameters.

Considering that we could not determine, for this pilot study, the number of dogs by statistical estimation but according to housing capacity the non-significant results on the effects of a HFD in dogs on the evolution of various parameters, especially on the intestinal microbiota must therefore be analyzed with caution.

## Conclusions

This study aimed to compare the effects of a HFD fed at maintenance energy requirements to those of the same diet at 150% maintenance on the fecal microbiota, colonic barrier, and metabolic and inflammatory status in dogs. Microbiota diversity was reduced only in the case of a HFD in excess and this was associated with greater changes in microbiota composition and a decrease in insulin sensitivity compared to the same diet fed at maintenance levels. No increase in endotoxemia nor in systemic inflammation was observed, which could have helped to explain the role the intestinal microbiota plays in the decrease in insulin sensitivity.

The transient alteration of the permeability of the intestinal barrier seems to have been caused by an alteration in the intestinal microbiota. The restoration of intestinal permeability could have been due to an increase in the expression of genes coding for proteins involved in the maintenance of the intestinal barrier. The investigation of the metagenome and fecal metabolome could provide a better understanding of the involvement of the microbiota in perturbations of the intestinal barrier and to metabolic damage.

## Data Availability Statement

Raw sequences were submitted to NCBI GenBank database under BioProject number PRJNA639131.

## Ethics Statement

The animal study was reviewed and approved by Royal Canin's Ethical Review Committee (090118-03) and the Ethics Committee of Pays-de-la-Loire (Apafis#13800).

## Author Contributions

JF, PN, and VL: conceptualization, methodology, and validation. AM, CP, and VL: formal analysis. AM, AA, JH, AD, JS, and VL: investigation. AM and CP: data curation. AM: writing—original draft preparation, visualization, and project administration. CP, KO, JH, AD, VB, JS, JF, PN, and VL: writing—review and editing. PN and VL: funding acquisition. All authors have read and agreed to the published version of the manuscript.

## Conflict of Interest

JF and VB were employed by Royal Canin SAS. The remaining authors declare that the research was conducted in the absence of any commercial or financial relationships that could be construed as a potential conflict of interest.

## Supplementary Material

Supplementary Figure 1 **(A)** Triglycerides (TG), **(B)** Total cholesterol, **(C)** HDL-cholesterol, during a feed-challenge test in dogs fed the high-fat diet at maintenance (HF-100; *n* = 8) and at 150% maintenance (HF-150; *n* = 8) for 8 weeks. Data are mean ± SEM.

Supplementary Table 1 Relative abundance of the fecal microbiome in dogs fed the high-fat diet at maintenance (HF-100; *n* = 8) and at 150% maintenance (HF-150; *n* = 16) for 8 weeks.

Supplementary Table 2 Digestibility at week 0 and week 8 for HF-100 and HF-150 groups. Values are mean ± SEM.
