# Supplementary figures and images for: Effects of High-Fat Diet at Two Energetic Levels on Fecal Microbiota, Colonic Barrier, and Metabolic Parameters in Dogs
Source: Front Vet Sci. 2020 Sep 25;7:566282. doi: 10.3389/fvets.2020.566282 (PMC7545960; doi:10.3389/fvets.2020.566282)

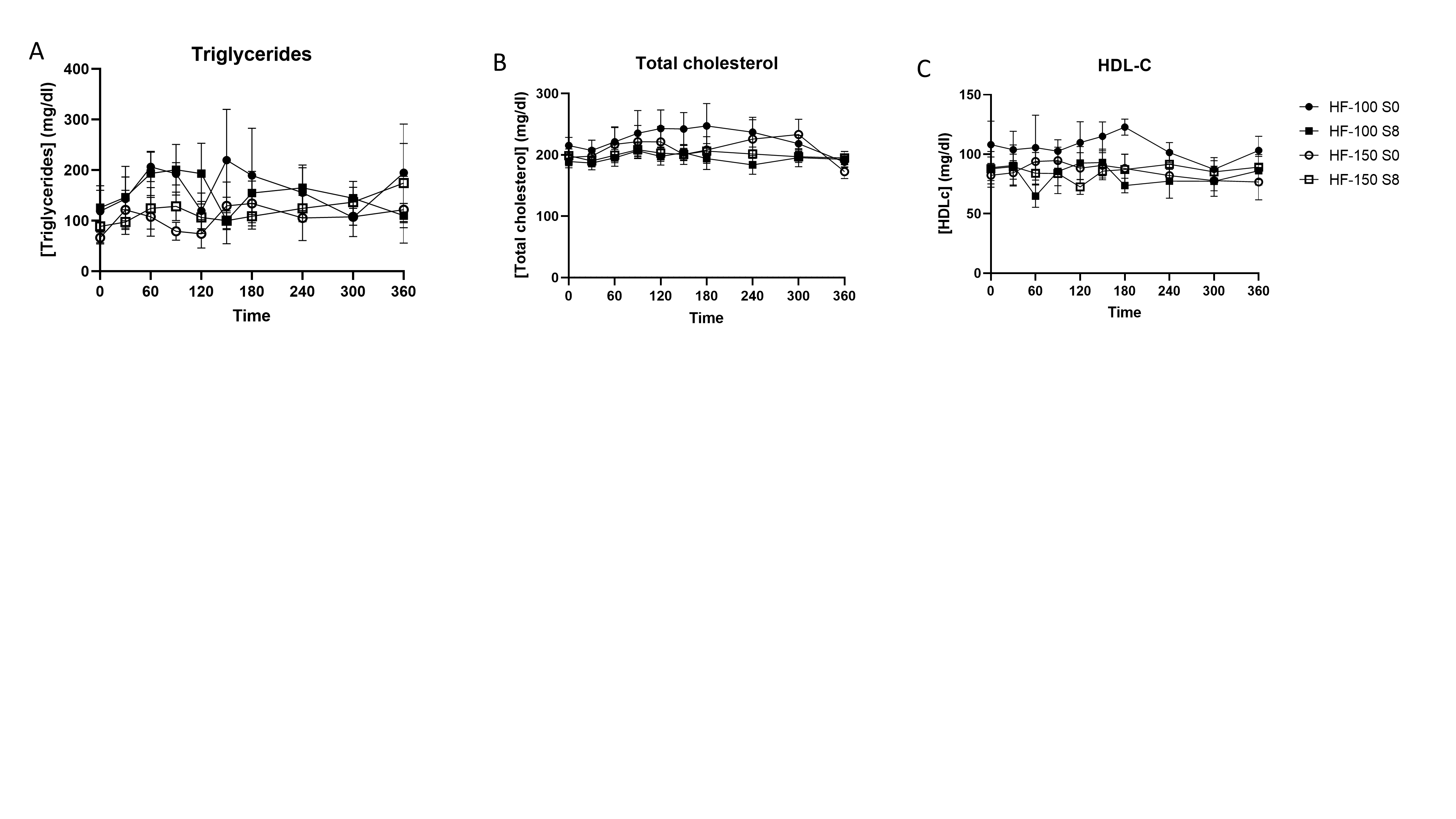

Supplement: Supplementary Figure 1 — (A) Triglycerides (TG), (B) Total cholesterol, (C) HDL-cholesterol, during a feed-challenge test in dogs fed the high-fat diet at maintenance (HF-100; n = 8) and at 150% maintenance (HF-150; n = 8) for 8 weeks. Data are mean ± SEM. [file Image_1.TIF]
